# A higher De Ritis ratio (AST/ALT) is a risk factor for progression in high-risk non-muscle invasive bladder cancer

**DOI:** 10.18632/oncotarget.27944

**Published:** 2021-04-27

**Authors:** Sachi Fukui-Kawaura, Takashi Kawahara, Yushi Araki, Reona Nishimura, Koichi Uemura, Kazuhiro Namura, Nobuhiko Mizuno, Masahiro Yao, Hiroji Uemura, Ichiro Ikeda

**Affiliations:** ^1^Department of Urology, Yokohama Minami Kyousai Hospital, Yokohama, Japan; ^2^Departments of Urology and Renal Transplantation, Yokohama City University Medical Center, Yokohama, Japan; ^3^Department of Urology, Yokohama City University, Graduate School of Medicine, Yokohama, Japan

**Keywords:** AST, ALT, De Ritis, high risk NMIBC

## Abstract

Background: High-risk non-muscle invasive bladder cancer (NMIBC) is thought to be associated with a higher risk of recurrence and progression. A recent study revealed that a high De Ritis ratio was a risk factor in some solid malignancies. This study examined the importance of the De Ritis ratio as a prognostic marker in high-risk NMIBC.

Materials and Methods: A total of 138 patients who were initially diagnosed with high-risk NMIBC between January 2012 to December 2016 were enrolled in this study. The criteria for the high-risk classification followed the EAU guidelines. The recurrence-free and progression-free survival of the higher and lower De Ritis ratio groups were compared. The cut-off value of the De Ritis ratio was set at 1.35, based on a receiver operator curve analysis.

Results: The median observation period was 50.3 months. Among these patients, 32 (23.1%) patients developed recurrent disease and 15 (10.9%) patients showed progression. A multivariate analysis revealed that non-BCG treatment was an independent risk factor for recurrence, and a higher De Ritis ratio was an independent risk factor for cancer progression.

Conclusions: The De Ritis ratio might be a risk factor for progression in high-risk NMIBC.

## INTRODUCTION

Bladder cancer is the eleventh most common malignant disease in the world, and non-muscle invasive bladder cancer (NMIBC) accounts for 75% of all bladder cancer cases [1]. Although NMIBC is treated by trans-urethral endoscopic surgery, high-risk NMIBC easily progresses to MIBC and shows a high rate of recurrence [2, 3].

The recommended standard treatments for high-risk NMIBC, which is defined by the NCCN and EAU guidelines, include intra-vesicle BCG or repeat TUR-Bt [4]. Although these are considered to be intensive treatments, the rates of recurrence and progression are still high. Thus, more detailed criteria are needed for the risk assessment.

The serum aspartate aminotransaminase (AST)/alanine aminotransaminase (ALT) ratio was firstly reported by De Ritis in 1957, since then this ratio has been called the De Ritis ratio [5]. ALT is only present in liver cells, while AST is present in heart, renal, brain, muscle, and liver cells [6]. A high De Ritis ratio was reported to be a poor prognostic marker in some solid malignancies [7]. In genitourinary cancer, a high De Ritis ratio was reported to be a poor prognostic marker in prostate, renal, and urothelial carcinoma. In other solid malignancies including breast and lung cancer, a high De Ritis ratio was also reported to be a poor prognostic marker.

This study examined the importance of the De Ritis ratio as a prognostic marker in high-risk NMIBC.

## RESULTS

The patients’ characteristics including age, urinary cytology, pathological T stage, CIS (or not), tumor size, number of tumors, tumor grade, repeat TUR (or not), and BCG instillation are shown in Table 1. The median (mean ± SD) age was 75 years (74.4 ± 9.8 years) and 109 patients (79.0%) were male. Higher De Ritis groups showed significantly higher age comparting to the lower De-Ritis group (*p* < 0.001). Among these patients 32 patients (23.2%) showed recurrence and 15 (10.9%) showed progression. The median (mean ± SD) observation period was 50.3 months (51.1 ± 28.6 months). The median recurrence-free survival time was 40.8 months (42.7 ± 28.2 months) and the median progression free survival time was 48.2 months (48.2 ± 29.2 months). At the time of the initial diagnosis, the rate of high-grade pTa was 25.4%, and the rate of pT1 was 50.0%. Thirty-four patients (24.6%) had CIS, 90 (65.2%) had multiple tumors, and 28 (20.3%) had tumors of ≥ 3 cm in diameter. Fifty-seven patients (41.3%) underwent repeat TUR-Bt and 112 patients (81.2%) received postoperative intra-vesical treatment, including BCG in 101 patients (73.1%) and pirarubicin in 11 patients (8.0%).

**Table 1:** Patients’ characteristics

		All (*n* = 138)	AST/ALT < 1.35 (*n* = 81)	AST/ALT ≦1.35 (*n* = 57)	*p* value
Age, yr	< 75	66 (47.8)	50 (61.7)	16 (28.0)	0.260
	75≦	72 (52.2)	31 (38.3)	41 (71.9)	
Sex	Female	29 (21.0)	14 (17.3)	15 (26.3)	0.201
	Male	109 (79.0)	67 (82.7)	42 (73.7)	
Urinary Cytology	I–III	50 (36.2)	33 (40.7)	17 (29.8)	0.308
	IV–V	79 (57.2)	45 (55.6)	34 (59.6)	
	Unknouwn	9 (6.5)	3 (3.7)	6 (10.5)	
T category	pT1	69 (50.0)	41 (50.6)	28 (49.1)	0.668
	CIS	34 (24.6)	17 (21.0)	17 (29.8)	
	HG pTa	35 (25.4)	23 (28.4)	12 (21.1)	
	Multiple+LG pTa+3 cm≦	0 (0.0)	0 (0.0)	0 (0.0)	
CIS	No	104 (75.4)	64 (79.0)	40 (70.2)	0.237
	Yes	34 (24.6)	17 (21.0)	17 (29.8)	
No. of Tumors	Single	44 (31.9)	29 (35.8)	15 (26.3)	0.208
	Multiple	90 (65.2)	49 (60.5)	41 (71.9)	
	Unknown	4 (2.9)	3 (3.7)	1 (1.8)	
Tumor size	< 3 cm	110 (79.7)	64 (79.0)	46 (80.7)	0.809
	3 cm≦	28 (20.3)	17 (21.0)	11 (19.3)	
Grade	Low	19 (13.8)	11 (13.6)	8 (14.0)	0.863
	High	115 (83.3)	69 (85.1)	46 (80.7)	
	Unknown	4 (2.9)	1 (1.2)	3 (5.3)	
second TUR	No	81 (58.7)	44 (54.3)	37 (64.9)	0.215
	Yes	57 (41.3)	37 (45.7)	20 (35.1)	
Single instillation		103 (74.6)	63 (77.8)	40 (70.2)	0.329
Additional Delayed Bladder Instillation					
	None	25 (18.1)	12 (14.8)	13 (22.8)	0.183
	Pirarubicin	11 (8.0)	6 (7.4)	5 (8.8)	
	BCG	101 (73.1)	63 (77.8)	38 (66.7)	
	unknown	1 (0.7)	0 (0.0)	1 (1.8)	
BCG Maintenance		11 (8.0)	9 (11.1)	2 (3.5)	0.106

**Figure 1:**
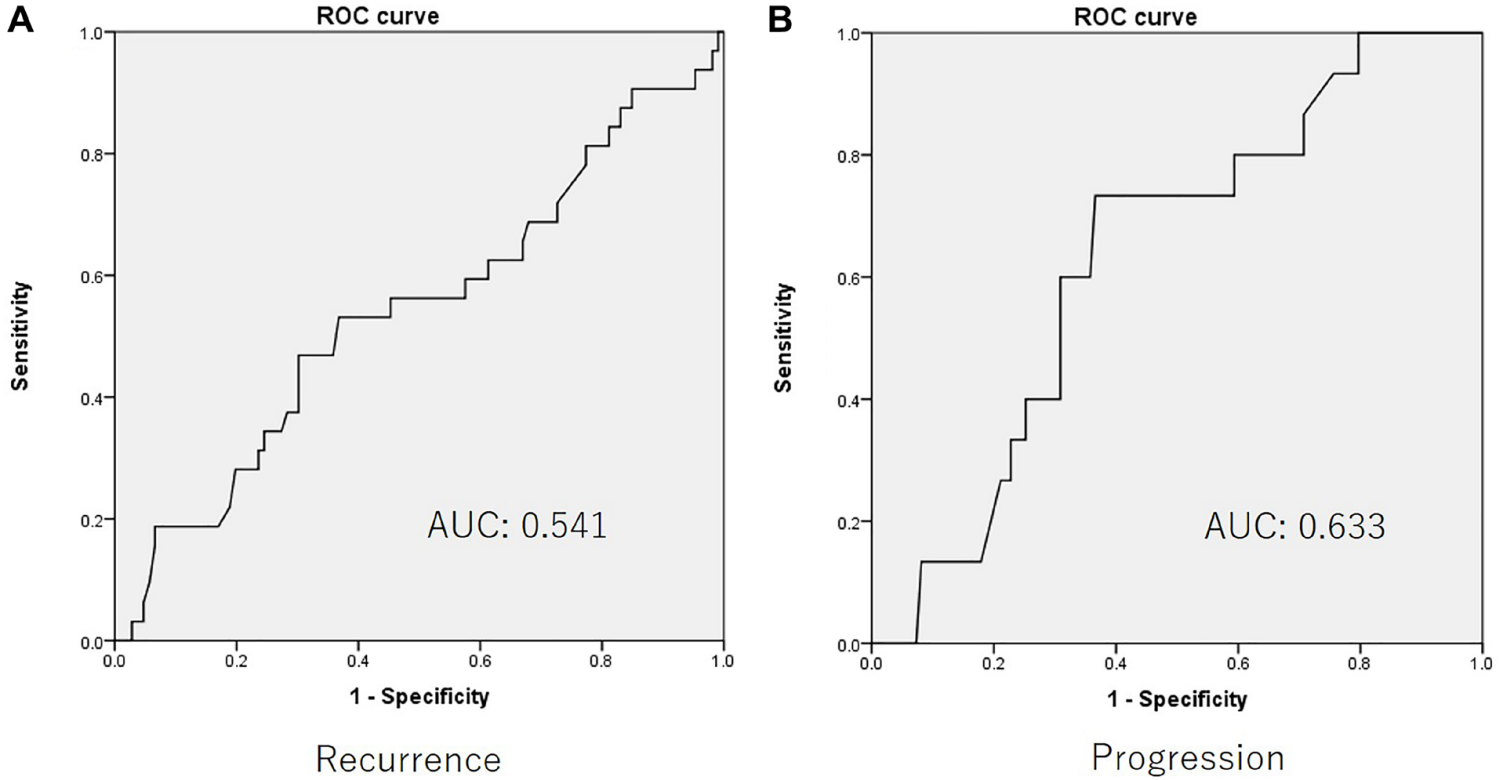
The receiver operator characteristic curve for (**A**) recurrence and (**B**) progression.

**Figure 2:**
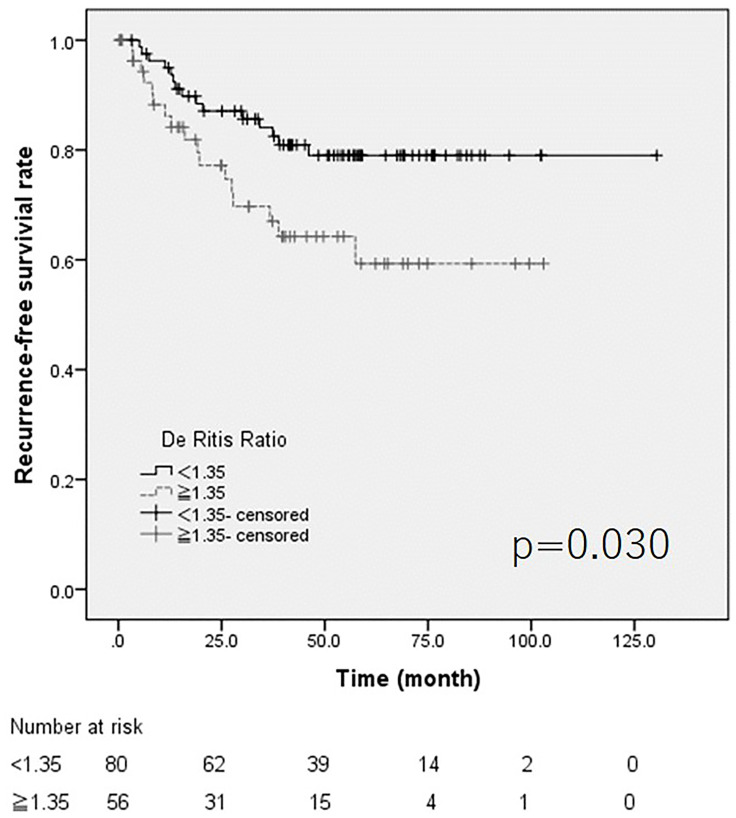
The Kaplan Meier curve for recurrence.

**Figure 3:**
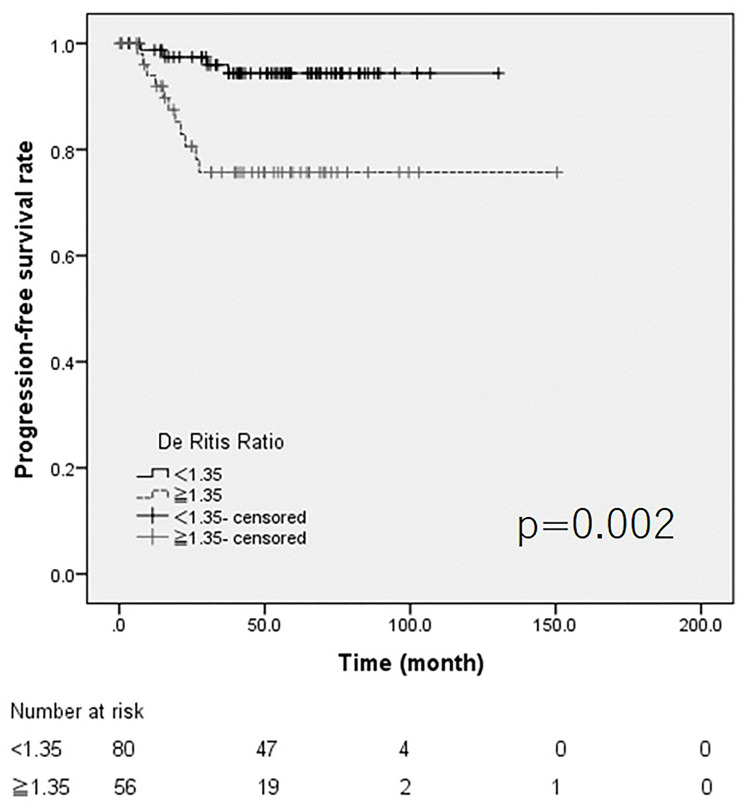
The Kaplan Meier curve for progression.

The cut-off value of the De Ritis ratio, which was selected based on the AUROC, was 1.35 (Figure 1A and 1B). Both ROCs for recurrence and progression showed same candidate cut-off points. Kaplan-Meier curve and a log-rank test revealed that the high De Ritis group showed poorer recurrence free and progression free survival (*p* = 0.030 and *p* = 0.002, respectively) (Figures 2 and 3). The multivariate analysis revealed that intra-vesicle BCG instillation was independent risk factor for recurrence (*p* = 0.002). A high De Ritis (≥ 1.35) ratio was the only independent risk factor for progression (HR = 5.558, 95% CI = 1.493–20.689, *p* = 0.01) (Tables 2 and 3).

**Table 2:** Multivariate analysis for recurrence

		HR	CI 95%	HR	*p*-value
			Lower	Upper	
Age, yr	< 75, 75≦	0.512	0.219	1.198	0.123
Sex	Female, Male	2.369	0.745	7.532	0.144
No. of tumors	Single, Multiple	1.254	0.547	2.871	0.593
Tumor size	< 3 cm, 3 cm≦	1.626	0.674	3.922	0.279
Single instillation	No, Yes	1.028	0.425	2.487	0.952
BCG	No, Yes	0.271	0.118	0.623	0.002
De Ritis Ratio	< 1.35, 1.35≦	2.232	0.996	5.003	0.051

**Table 3:** Multivariate analysis for progression

		HR	CI 95%	HR	*p*-value
			Lower	Upper	
Age, yr	< 75, 75≦	0.504	0.141	1.8	0.292
sex	Female, Male	4.537	0.528	39.02	0.168
No. of tumors	Single, Multiple	1.405	0.372	5.304	0.616
Tumor size	< 3 cm, 3 cm≦	1.941	0.575	6.548	0.285
Single instillation	No, Yes	1.246	0.327	4.742	0.747
BCG	No, Yes	0.568	0.158	2.037	0.385
De Ritis Ratio	< 1.35, 1.35≦	5.558	1.493	20.689	0.011

## DISCUSSION

In this study of patients with NMIBC, the high De Ritis ratio group showed higher rates of recurrence and progression. In the EORTC trial, among patients with high-risk NMIBC, the rate of recurrence within 5 years was approximately 80%, while the rate of progression within 5 years was approximately 50% [2]. In the CUETO trial, intra-vesicle BCG instillation reduced the rates of recurrence and progression in both intermediate- and high-risk NMIBC [3]. The present study also demonstrated the effectiveness of BCG instillation in reducing recurrence in high-risk NMIBC, although patients with high-risk NMIBC who received BCG treatment showed higher rates of recurrence and progression rate. Thus, the De Ritis ratio might be clue to identifying cases that would benefit from additional treatment, including total cystectomy.

The AST/ALT ratio, which is also known as the De Ritis ratio, was first reported by De Ritis in 1957 [5]. ALT is an enzyme that only exists in hepatic cells, while AST is present in the cells of the liver, kidney, brain, muscle, and other organs [6]. The De Ritis ratio was initially used as a marker of viral hepatitis; however, it is now used as a prognostic marker for various diseases, including hepatic cell carcinoma (HCC), breast cancer, renal cell carcinoma, testicular tumor, and urothelial carcinoma [8–12]. Although the detailed mechanism is still unknown, Li-xian Zhang et al. reported that a higher De Ritis ratio was associated with a poor prognosis in HCC. AST contains mitochondria, while ALT does not. One possible explanation for the association between a higher De Ritis ratio and a poor prognosis in HCC is that liver dysfunction due to a decrease in mitochondria may increase the serum AST level [8]. Recently Yuk et al. reported that a higher De Ritis ratio was a poor prognostic factor in patients undergoing total cystectomy [13]. The present study is the first to analyze the association between the De Ritis ratio and the prognosis in high-risk NMIBC and found a similar relationship. Our results suggested that for high-risk NMIBC patients with higher De Ritis ratio might consider nao-adjuvant systemic chemotherapy in addition to total cystectomy.

Malignant disease uses the glycolysis system or malate-aspartate shuttle as an energy metabolic pathway [14, 15]. Both AST and ALT carry carbohydrates and are protein metabolic pathways. ALT is involved in the glucose-alanine cycle and AST is a metabolic pathway of the malate-aspartate shuttle in mitochondria [6]. AST also plays an important role in glucose metabolism [6]. Based on these mechanisms, high tumor progression activity may increase tumor metabolism and result in an increase in the AST/ALT ratio.

The present study is associated with several limitations. First, the study was retrospective in nature. Second, the sample size was relatively small. To reveal the usefulness of the De Ritis ratio as a biomarker, a longer-term study of a larger population with a prospective design should be performed. Third, this study showed higher age in higher De Ritis group. No previous study showed the correlation between AST/ALT value and age. Thus, further study is needed.

## MATERIALS AND METHODS

A total of 138 patients who were initially diagnosed with high-risk NMIBC in Yokohama Minami Kyousai Hospital (Yokohama, Japan) between 2012 to 2016 were included in this study. The institutional review board of Yokohama Minami Kyousai Hospital approved this study (IRB No: 1-20-2-63). High-risk NMIBC was defined according to the EAU guidelines as follows: 1) pathological T1, 2) carcinoma *in situ* (CIS), 3) pathological high-grade urothelial carcinoma, or 3) multiple sites with low-grade and pathological Ta with a maximum diameter of ≥ 3 cm. Some of the patients (*n* = 57, 41.3%) underwent repeat TUR-Bt, while 103 patients (74.6%) received 6 courses or more of intra-vesical chemotherapy using pirarubicin (30 mg in 15 min) 10 times per 6 months and intra-vesical BCG (80 mg in 60 min; Immunobladder, Japan BCG Lab, Tokyo, Japan) once a week. The De Ritis ratio was calculated at the time of preoperative examination. Follow-up examinations included cystoscopy and urinary cytology performed every 3 months until two years from the initial diagnosis.

### Statistical analyses

The patients’ characteristics and preoperative factors were analyzed by the Mann-Whitney *U* test using the SPSS software program (SPSS 22.0, Chicago, IL, USA) and the Graph Pad Prism software program (Graph Pad Software, La Jolla, CA, USA). Candidate cut-off points were identified using the area under receiver operator curve (AUROC). The survival duration was defined as the time between the date of the initial pathological diagnosis and the date of tumor recurrence or progression (muscle invasiveness or metastasis). A log-rank test was performed for comparisons between the high and low De Ritis ratio groups. A Cox regression analysis was performed as a multivariate analysis. *P* values of < 0.05 were considered to indicate statistical significance.

## CONCLUSIONS

The De Ritis ratio might be a risk factor for progression in high-risk NMIBC.

## Abbreviations

NMIBC: non-muscle invasive bladder cancer
AST: aspartate aminotransaminase
ALT: alanine aminotransaminase
CIS: carcinoma *in situ*
HCC: hepatic cell carcinoma
AUROC: area under receiver operator curve
